# Disseminated paracoccidioidomycosis in a black lion tamarin (*Leontopithecus chrysopygus*) confirmed by histopathology, molecular diagnostics and phylogenetic analysis of DNA from FFPE tissues

**DOI:** 10.1007/s11259-026-11455-2

**Published:** 2026-08-08

**Authors:** Isabelle Dias de Oliveira, Isabella Galesi Tallach, Jamile Macedo Garcia, Gabrielle Fernanda Pereira da Silva Gagliotti, Jessica de Brito Ferreira Nascimento, Camila Santos da Silva Ferreira, Silvana Mello Pereira da Silva, Clarice Ribeiro Roque de Assumpção, Juliana Possatto Fernandes Takahashi, Julia de Carvalho, Mayara Grego Caiaffa, Daniela Etlinger-Colonelli, Natália Coelho Couto de Azevedo Fernandes, Leonardo José Tadeu de Araújo

**Affiliations:** 1 Programa de Pós Graduação em Ciências da Saúde do Instituto de Assistência Médica ao Servidor Público Estadual – IAMSPE, São Paulo, Brazil; 2 Centro de Patologia, Instituto Adolfo Lutz, São Paulo, Brazil; 3 Programa de Pós-Graduação em Ciências, Coordenadoria de Controle de Doenças, Secretaria de Estado de Saúde de São Paulo, São Paulo, Brazil; 4 Faculdade de Ciências Médicas da Santa Casa de São Paulo, São Paulo, Brazil; 5 Departamento de Conservação de Fauna Silvestre – Secretaria de Meio Ambiente, Infraestrutura e Logística do Estado de São Paulo, São Paulo, Brazil

**Keywords:** Paracoccidioidomycosis, *Leontopithecus*, Primates, One Health, Molecular Epidemiology

## Abstract

**Background:**

Paracoccidioidomycosis (PCM) is a systemic mycosis endemic to Latin America, primarily affecting humans. Reports in non-human primates are rare and provide valuable insight into the environmental circulation of *Paracoccidioides* spp. and the interface between animal and human infection.

**Case report:**

We describe a case of disseminated PCM in a 13-year-old black lion tamarin (*Leontopithecus chrysopygus*) from São Paulo, Brazil. The animal presented neurological signs and marked clinical deterioration, progressing to death within 48 h of the onset of neurological signs. Histopathological analysis of multiple organs revealed granulomatous inflammation associated with multiple budding yeast-like organisms consistent with *Paracoccidioides* spp., confirmed by Grocott staining. Molecular analysis of formalin-fixed, paraffin-embedded (FFPE) tissue by polymerase chain reaction (PCR) detected *Paracoccidioides* spp., and the resulting Sanger sequences clustered with reference sequences of the *Paracoccidioides brasiliensis* complex in the ITS-based phylogenetic analysis.

**Conclusion:**

This case demonstrates severe disseminated PCM in an endangered Neotropical primate and highlights the diagnostic value of combining histopathology and molecular assays in FFPE samples for etiological confirmation of fungal infections.

## Introduction

Paracoccidioidomycosis (PCM) is a systemic mycosis endemic to Latin America, caused by thermodimorphic fungi of the genus *Paracoccidioides*, with high prevalence in Brazil, which accounts for approximately 80% of reported cases worldwide (Peçanha et al. 2022). Infection is typically acquired via inhalation of airborne propagules, followed by primary pulmonary infection and potential hematogenous and lymphatic dissemination to multiple organs (Ferreira 2009). In formalin-fixed, paraffin-embedded (FFPE) specimens, etiological confirmation can be achieved through the combined use of histopathology, special stains, and molecular methods (Pinheiro et al. 2021, 2023).

*Paracoccidioides* spp. infect a broad range of mammalian hosts, reflecting their environmental niche and circulation in nature. This shared environmental exposure underscores ecological links between environmental, animal, and human health. Accordingly, infections in wild animals have been used as indirect indicators of environmental presence and as valuable tools for eco-epidemiological investigations (Albano et al. 2014; de Oliveira et al. 2024). In this context, we report a case of disseminated infection caused by *Paracoccidioides* spp. in a black lion tamarin (*Leontopithecus chrysopygus*), confirmed by histopathology, Grocott staining, and polymerase chain reaction (PCR) performed on FFPE material.

## Case presentation

A 13-year-old adult male black lion tamarin (*Leontopithecus chrysopygus*), born at the former São Paulo Zoological Park Foundation (FPZSP) in 2012 and transferred in 2015 to the Wildlife Conservation Department of the State of São Paulo (CECFAU), Brazil, was evaluated. Long-term retrospective records concerning the sanitary and health management of the colony were not available for evaluation. On clinical examination, initially, the animal presented a markedly poor body condition score (BCS 1/5), weighing 418 g, with selective appetite and mild apathy, progressing to nystagmus and head spasms. Medical intervention was immediately instituted; the tamarin was transferred to an incubator in the intensive care unit, received supportive care, and was administered dipyrone. Although the nystagmus and head spasms ceased after initial treatment, the animal was found dead the following day, within 48 h of the onset of neurological signs. An ultrasonographic examination performed in 2024, before the animal’s death in November 2025, had revealed concurrent nephropathy, enteritis, and hepatopathy.

Complete necropsy was performed with main gross findings involving the kidneys and liver. The kidneys were reddish, with multifocal blackened areas, smooth and non-adherent capsule. On cut section, there was mild loss of corticomedullary distinction and cysts. The liver showed accentuated lobular pattern, with wine-colored areas and blackened margins; on sectioning, it was firm with irregular texture. The gallbladder was mildly distended with dark content.

Multiple formalin-fixed organ samples (including liver, spleen, kidney, mesenteric lymph node among others) were submitted to the Instituto Adolfo Lutz for Yellow Fever Surveillance Program.

**Fig. 1:**
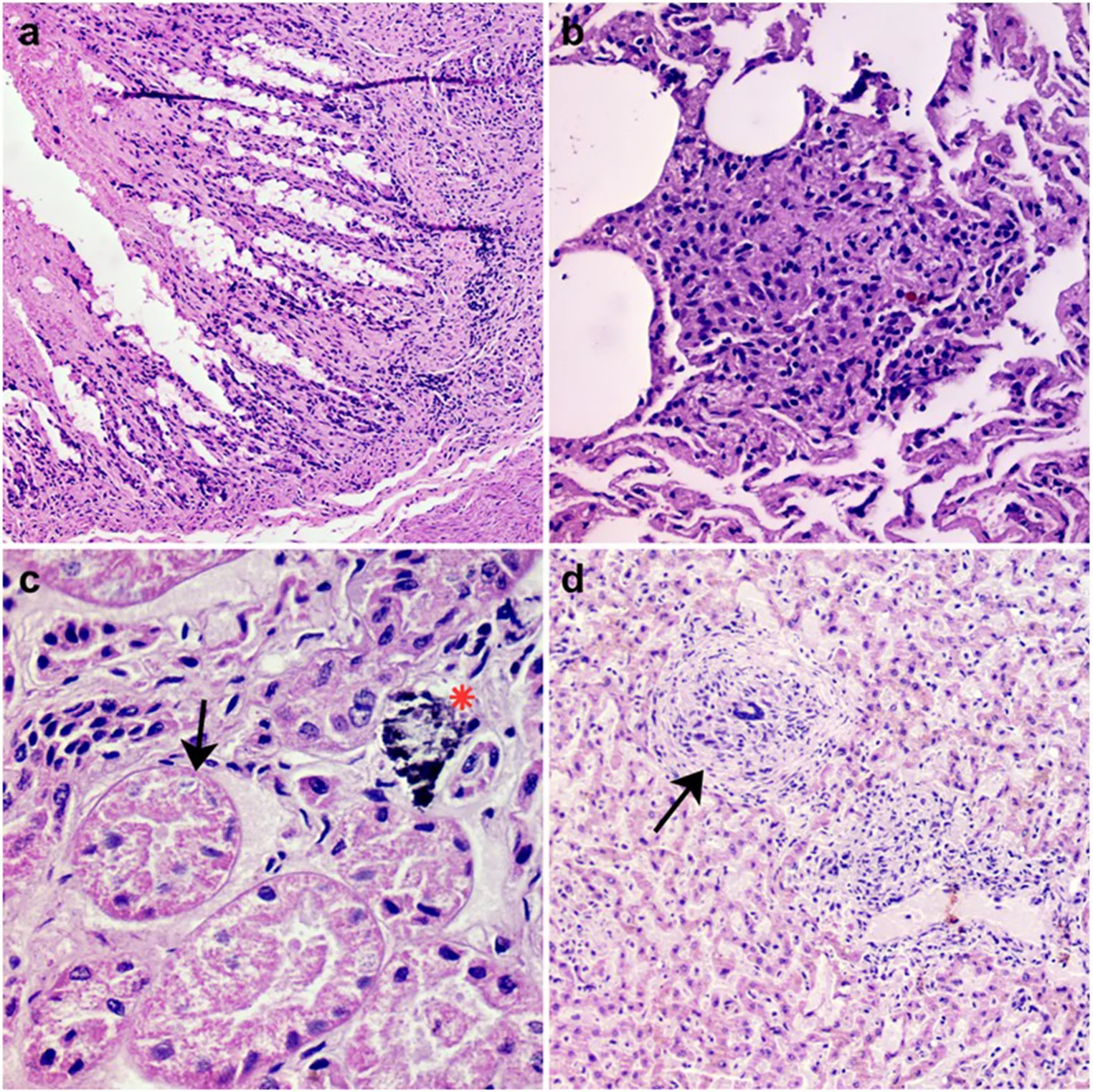
Disseminated PCM in a black lion tamarin (*Leontopithecus chrysopygus*). **(a)** Intestine: marked enteritis. H&E, x10. **(b)** Lung: mononuclear inflammatory infiltrate. H&E, x20. **(c)** Kidney: acute tubular injury (arrow) with focal mineralization (asterisk). H&E, x20. **(d)** Liver: lobular mixed hepatitis with granuloma formation. H&E, x10

**Fig. 2:**
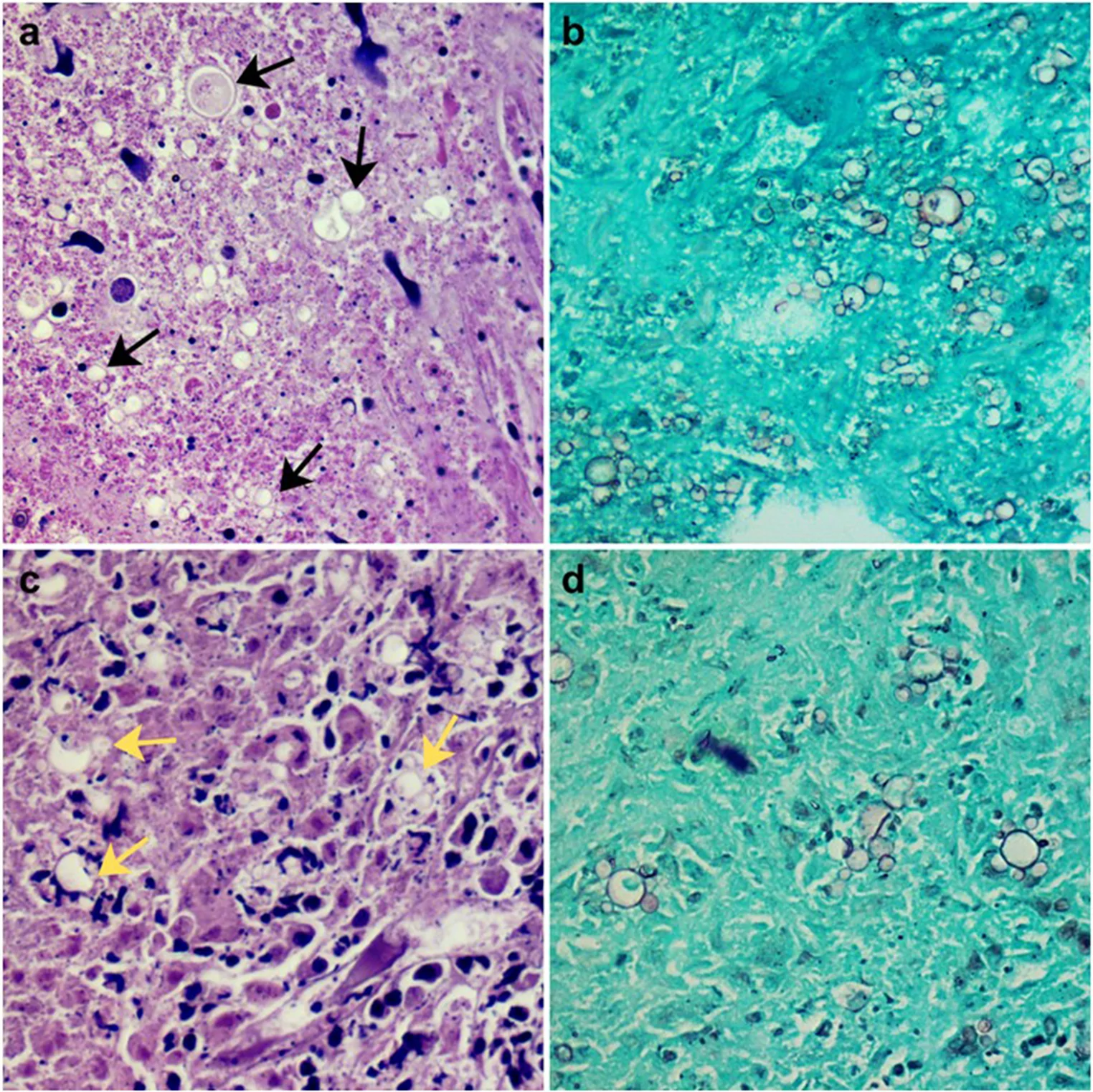
Histopathological features of *Paracoccidioides* spp. infection in a black lion tamarin (*Leontopithecus chrysopygus*). **(a)** Intestine: numerous yeast-like organisms dispersed within the tissue. H&E, x20. **(b)** Intestine: thick-walled yeast cells with multiple budding forms. GMS, x20. **(c)** Mesenteric lymph node: yeast-like organisms associated with macrophagic infiltrate. H&E, x40. **(d)** Mesenteric lymph node: budding yeast exhibiting characteristic “pilot wheel” morphology of *Paracoccidioides* spp. GMS, x40

Histopathology of the intestine revealed marked multifocal to coalescing enteritis (Fig. 1a), associated with numerous large, spherical to oval yeast-like organisms with thick walls and multiple budding (Fig. 2a), also highlighted by Grocott–Gomori methenamine silver (GMS) staining (Fig. 2b). The mesenteric lymph node showed marked macrophagic lymphadenitis with multinucleated giant cells, granuloma formation, and similar yeast-like organisms (Fig. 2c), confirmed by GMS staining (Fig. 2d). The lungs exhibited mild multifocal alveolar edema and mild multifocal mononuclear inflammation (Fig. 1b). The kidneys showed diffuse, marked acute tubular injury of proximal convoluted tubules, with mild multifocal mineralization and mild to moderate autolysis (Fig. 1c). The liver showed mild multifocal lobular mixed hepatitis with multinucleated giant cells, hepatic granuloma formation, mild microvesicular steatosis, and diffuse hemosiderosis (Fig. 1d). In the remaining organs, mild to moderate autolysis predominated. Gram staining did not reveal bacteria in multiple organs, and Perls’ staining confirmed iron pigment deposition in the liver.

DNA was extracted from FFPE tissue sections using the QIAamp DNA FFPE Tissue Kit (QIAGEN), according to the manufacturer’s instructions. Molecular investigation included real-time PCR (qPCR) using the primers *Paracoco-F* (5’-CGTATGGGGCTTCGTCACAC-3’) and *Paracoco-R* (5’-CCCTACCTGATCCGAGGTCAAC-3’), together with the Paracoco-Fam probe (5’-CCCGCCGGGGTCGATGAGT-3’), originally described in a multiplex assay for detection of the *Paracoccidioides brasiliensis* complex. In addition, conventional PCR (cPCR) was performed using the Paracoco primer set for detection of *Paracoccidioides* spp., as well as the *PLU* primer set (*PLU-F*: 5’-CTTCATGGCGCCCAAGGACTT-3’ and *PLU-R*: 5’-GACTCCGCTAAGTCTGTCCTTAGGC-3’) for specific screening of *Paracoccidioides lutzii*. Amplification was detected in the Paracoco assay, whereas the PLU assay yielded a non-detectable result (Pinheiro et al. 2021, 2023). The cPCR product was submitted to Sanger sequencing for further confirmation. The resulting sequences were deposited in GenBank under accession numbers PZ255820 and PZ255821.

**Fig. 3:**
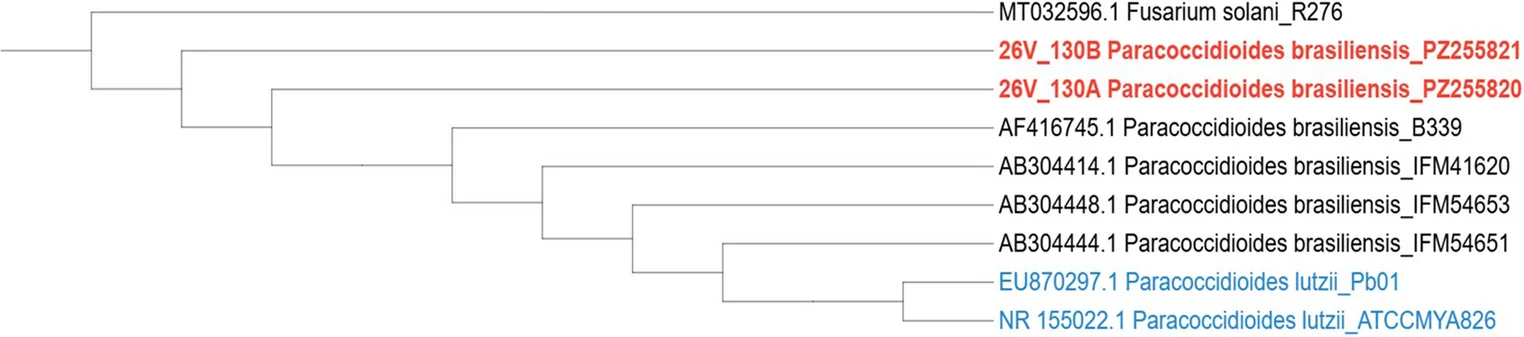
Maximum-likelihood phylogenetic tree based on a 143-bp ITS-derived region of *Paracoccidioides* spp. Study sequences 26V_130A and 26V_130B are highlighted in red. The tree was inferred using IQ-TREE v2 under the K3Pu + F substitution model. Branch support was assessed using 1,000 ultrafast bootstrap and 1,000 SH-aLRT replicates. *Fusarium solani* was used as the outgroup

For phylogenetic analysis, the study sequences were compared with reference sequences of the *Paracoccidioides* species complex retrieved from GenBank. A maximum-likelihood tree was inferred using IQ-TREE v2 under the K3Pu + F substitution model, selected by ModelFinder according to the Bayesian information criterion. Branch support was assessed using 1,000 ultrafast bootstrap and 1,000 SH-aLRT replicates, and *Fusarium solani* was used as the outgroup. The resulting topology is shown in Fig. 3.

## Discussion

PCM is the most important systemic mycosis in Latin America, associated with substantial morbidity and mortality in humans in endemic regions. In Brazil, the mortality rate is approximately 3.18 deaths per million inhabitants (Peçanha et al. 2022).

The molecular findings suggesting an association with the *Paracoccidioides brasiliensis* complex should be considered within the regional epidemiological context. The state of São Paulo is an endemic area for PCM, with heterogeneous distribution associated with edaphoclimatic conditions favorable to fungal persistence (Pinto et al. 2012; Kamikawa et al. 2012). Environmental and geoclimatic factors may influence the spatial distribution of PCM in endemic regions (Fabris et al. 2024). Although no direct evidence of endemicity exists at the institution where the animal was housed, the animal spent its early life at an institution situated within the Parque Estadual das Fontes do Ipiranga, an urban fragment of Atlantic Forest with ecological conditions compatible with the environmental niche of *Paracoccidioides* spp. Subsequently, it was transferred to the Wildlife Conservation Center of the State of São Paulo (CECFAU), situated in an inland region with Cerrado-related characteristics. Considering that PCM may remain latent for several years before clinical manifestation, the exact site of exposure could not be determined. Increasing reports in individuals without classical rural exposure suggest that urban forest fragments may represent potential sites of exposure (Benard et al. 2001).

Non-human primates may serve as valuable environmental sentinels because they share habitats and routes of exposure to environmentally acquired pathogens with humans and other mammals. The detection of PCM in these animals may indicate the local environmental circulation of *Paracoccidioides* spp., particularly in areas affected by forest fragmentation, environmental disturbance, and urban expansion. Therefore, surveillance of clinical disease and fungal exposure in free-ranging and captive primates may contribute to identifying potential areas of exposure and improving knowledge of the ecological distribution of the fungus. In this context, the occurrence of PCM in an endangered species such as the black lion tamarin reinforces the importance of integrating wildlife health into One Health surveillance strategies (Albano et al. 2014; de Macedo et al. 2020; de Souza Scramignon-Costa et al. 2021; de Oliveira et al. 2024). The inclusion of PCM as a notifiable disease in the state of São Paulo (Resolution SS No. 88/2024, São Paulo State Health Secretariat) further underscores its relevance as a public health concern in endemic areas.

While callitrichids are predominantly arboreal, their specific foraging behavior may explain how they come into contact with geotropic fungi. *L. chrysopygus* frequently searches for insects and larvae in the lower strata of the forest, probing tree cavities, stripping loose bark, and inspecting fallen decomposing logs, with occasional descents to the ground (Passos and Keuroghlian 1999). Since *Paracoccidioides* spp. are found in the soil and organic matter, this constant handling of substrates could release and aerosolize fungal propagules, facilitating infection via inhalation.

Clinical PCM has been reported in domestic animals, particularly dogs, including cases of systemic disease and generalized lymphadenomegaly (Ricci et al. 2004; de Farias et al. 2011). Among non-human primates, disseminated PCM has been described in a captive western black-handed tamarin (*Saguinus niger*) (de Oliveira et al. 2024). The limited number of available clinical reports suggests that New World primates may be susceptible to severe and disseminated PCM. This susceptibility may be associated with an ineffective granulomatous response, allowing rapid lymphohematogenous dissemination (Corte et al. 2007; Richter 2012). Consistently, the present case exhibited gastrointestinal involvement and subsequent lymphoid spread.

The pathogenesis observed in the present case resembles the acute/subacute human form, characterized by lymphoid tropism, visceral dissemination, poorly organized granulomas, and abundant multiple budding yeast-like organisms, present both intracellularly and extracellularly (Lowenstine et al. 2018; Carneiro et al. 2010). In humans, this clinical presentation is driven by a Th2-mediated immune response that generates a permissive tissue environment, which fails to activate macrophages effectively and prevents the formation of compact, protective granulomas (Valdovinos Zaputovich and Giusiano 2025). The histopathological similarities observed in the present case may suggest an immune response analogous to the Th2-associated pattern described in the acute/subacute human form. However, the immune profile of the tamarin was not directly investigated.

Given the fastidious nature of the agent and the limited sensitivity of environmental isolation from soil, molecular confirmation was essential. The combination of histopathological identification of characteristic yeast forms with qPCR and conventional PCR applied to FFPE tissue enabled reliable etiological confirmation despite autolysis, highlighting the value of archival material for molecular epidemiology (Pinheiro et al. 2021, 2023).

Phylogenetic analysis based on the ITS region showed that the sequences obtained in this study (26 V 130 A and 26 V 130B) were molecularly consistent with the *Paracoccidioides brasiliensis* complex, showing 96% identity to reference sequences available in GenBank. Together with the positive Paracoco assay and the non-detectable result obtained with the PLU-specific assay, these findings support an association with the *P. brasiliensis* complex.

However, this interpretation should be made with caution. The phylogenetic analysis was based on a short, 143-bp ITS-derived fragment containing a limited number of phylogenetically informative sites. In addition, DNA fragmentation associated with FFPE tissues may have affected sequence length and quality. Therefore, although the molecular findings were consistent with the *P. brasiliensis* complex, the ITS region alone did not provide sufficient resolution for definitive species-level discrimination among closely related members of the complex. Multilocus sequencing using additional genetic markers may provide greater taxonomic resolution (de Oliveira et al. 2023).

Finally, the severe systemic involvement and marked lymphohematogenous dissemination observed in this case resemble the acute/subacute juvenile form described in humans (Valdovinos Zaputovich and Giusiano 2025; da Cruz et al. 2021), suggesting that callitrichids may be susceptible to severe and disseminated PCM. This report highlights the relevance of wildlife surveillance in the early detection of pathogens of public health concern while providing insight into their distribution, host–environment interactions, and associated histomorphological patterns.

## Data Availability

The nucleotide sequences generated in this study were deposited in GenBank under accession numbers PZ255820 and PZ255821. Additional data supporting the findings of this study are available from the corresponding author upon reasonable request.
